# Efficient Detection of Novel Nuclear Markers for Brassicaceae by Transcriptome Sequencing

**DOI:** 10.1371/journal.pone.0128181

**Published:** 2015-06-10

**Authors:** Reinhold Stockenhuber, Stefan Zoller, Rie Shimizu-Inatsugi, Felix Gugerli, Kentaro K. Shimizu, Alex Widmer, Martin C. Fischer

**Affiliations:** 1 Institute of Evolutionary Biology and Environmental Studies, University of Zurich, Zurich, Switzerland; 2 Genetic Diversity Centre, ETH Zurich, Zurich, Switzerland; 3 WSL Swiss Federal Research Institute, Birmensdorf, Switzerland; 4 Institute of Integrative Biology, ETH Zurich, Zurich, Switzerland; National Institute of Plant Genome Research (NIPGR), INDIA

## Abstract

The lack of DNA sequence information for most non-model organisms impairs the design of primers that are universally applicable for the study of molecular polymorphisms in nuclear markers. Next-generation sequencing (NGS) techniques nowadays provide a powerful approach to overcome this limitation. We present a flexible and inexpensive method to identify large numbers of nuclear primer pairs that amplify in most Brassicaceae species. We first obtained and mapped NGS transcriptome sequencing reads from two of the distantly related Brassicaceae species, *Cardamine hirsuta* and *Arabis alpina*, onto the *Arabidopsis thaliana* reference genome, and then identified short conserved sequence motifs among the three species bioinformatically. From these, primer pairs to amplify coding regions (nuclear protein coding loci, NPCL) and exon-primed intron-crossing sequences (EPIC) were developed. We identified 2,334 universally applicable primer pairs, targeting 1,164 genes, which provide a large pool of markers as readily usable genomic resource that will help addressing novel questions in the Brassicaceae family. Testing a subset of the newly designed nuclear primer pairs revealed that a great majority yielded a single amplicon in all of the 30 investigated Brassicaceae taxa. Sequence analysis and phylogenetic reconstruction with a subset of these markers on different levels of phylogenetic divergence in the mustard family were compared with previous studies. The results corroborate the usefulness of the newly developed primer pairs, e.g., for phylogenetic analyses or population genetic studies. Thus, our method provides a cost-effective approach for designing nuclear loci across a broad range of taxa and is compatible with current NGS technologies.

## Introduction

For decades, evolutionary biologists have relied on a limited set of marker regions for DNA sequencing-based studies in plant population genetics, phylogenetics and phylogeography. Most often, organellar DNA [1] and nuclear ribosomal DNA (nrDNA) regions [2,3] have been used for these purposes. The widespread use of these marker regions is primarily a consequence of the availability of conserved primers for their amplification in a wide range of species, e.g. for the chloroplast genes *rbc*L [4], *mat*K [5] and for intergenic spacers, such as plastid *trn*L-F [6] and nrDNA internal transcribed spacer (ITS) regions [7]. Uniparental inheritance, single-locus origin and low mutation rates, in the case of organellar DNA, allow direct sequencing of PCR products because no heterozygosity is expected in these regions. For nrDNA, concerted evolution among gene family members reduces heterogeneity and facilitates direct sequencing of PCR products [2,8].

Despite their widespread use, organellar genomes have several disadvantages for evolutionary studies. Uniparental inheritance and lack of recombination (but see [9,10]), as well as low mutation rates in plants [8,11] present major limitations for inferring evolutionary history, because even if multiple markers are sequenced, they reflect variation at only a single locus and additionally lack information about one of the parental species (i.e. the pollen donor) in hybrid or in polyploid species [12–14]. Concerted evolution of multi-copy nrDNA markers, such as the internal transcribed spacer regions (ITS), may produce pseudogenes [15], remain incomplete [16] or lead to the loss of one parental copy in hybrids [17], which may also bias results in evolutionary studies. The limitations of these markers strongly suggest that evidence from multiple and unlinked nuclear markers, which are not affected by these limitations, should be used. Using such data may provide improved resolution and phylogenetic congruence among different loci may indicate that the phylogeny represents the underlying species history, as shown e.g. by Fink *et al* [14].

In addition to DNA sequencing, other types of molecular markers have also been used for multilocus analyses, including microsatellites and amplified fragment length-polymorphisms (AFLPs). Their specific advantages and disadvantages have been thoroughly reviewed elsewhere (see[14,18,19]). In general, these markers cannot easily be compared among distantly related species, as is done in many phylogenetic or comparative phylogeographic studies, or harness the information content of nucleotide variation, and therefore do not alleviate the need for multiple, independent DNA sequence-based markers. Moreover, nuclear DNA markers also allow addressing more complex evolutionary questions [20,21], for instance the detection of hybrid speciation [13,22,23] or rapid radiation events [12,14].

In the age of next-generation sequencing (NGS), multiple approaches can be taken for characterizing low-copy nuclear markers in a chosen group of organisms. Mining of genome sequence data, for example, has proven useful for the identification of shared single-copy nuclear genes in model organisms of the angiosperms [24]. It has been shown, that the merging of newly generated and publicly available sequence information allows to design primer pairs for closely related taxa [25,26], as well as for highly diverged groups [27–31]. However, for many study species or groups, no public datasets are available and existing data may be mislabeled [32] or erroneous [33]. Hence, it may be most valuable to generate *de-novo* sequence data, in combination with suitable reference genomes and the development of a dedicated bioinformatics workflow for primer or probe design, for characterizing multiple low-copy nuclear gene markers for population genetic, phylogenetic or phylogeographic analyses in a taxonomic group of interest.

We selected the mustard family (Brassicaceae) to evaluate novel approaches for the genome-wide characterization of nuclear gene markers and efficient primer design. In addition, we provide a database of the newly developed nuclear markers that are ready to use for a broad range of taxa. The Brassicaceae are ideally suited as a study group because multiple high-quality reference genomes for the model species *Arabidopsis thaliana* are available ([34], http://arabidopsis.org). Furthermore, phylogenetic relationships in the mustard family have been extensively studied using a range of markers, including several nuclear genes (e.g. [35–38]).

The main goal of the present study was to characterize low-copy nuclear gene markers on a genome-wide scale in the mustard family. To reach this goal, we first developed a powerful primer design approach for the amplification of markers across this phylogenetically and ecologically highly diverse Brassicaceae family. Second, we established a large database of ready to use nuclear Brassicaceae markers. And finally we validated the suitability of a subset of markers for PCR amplification, Sanger sequencing and phylogenetic reconstruction to highlight their usefulness.

## Material and Methods

**Fig 1:**
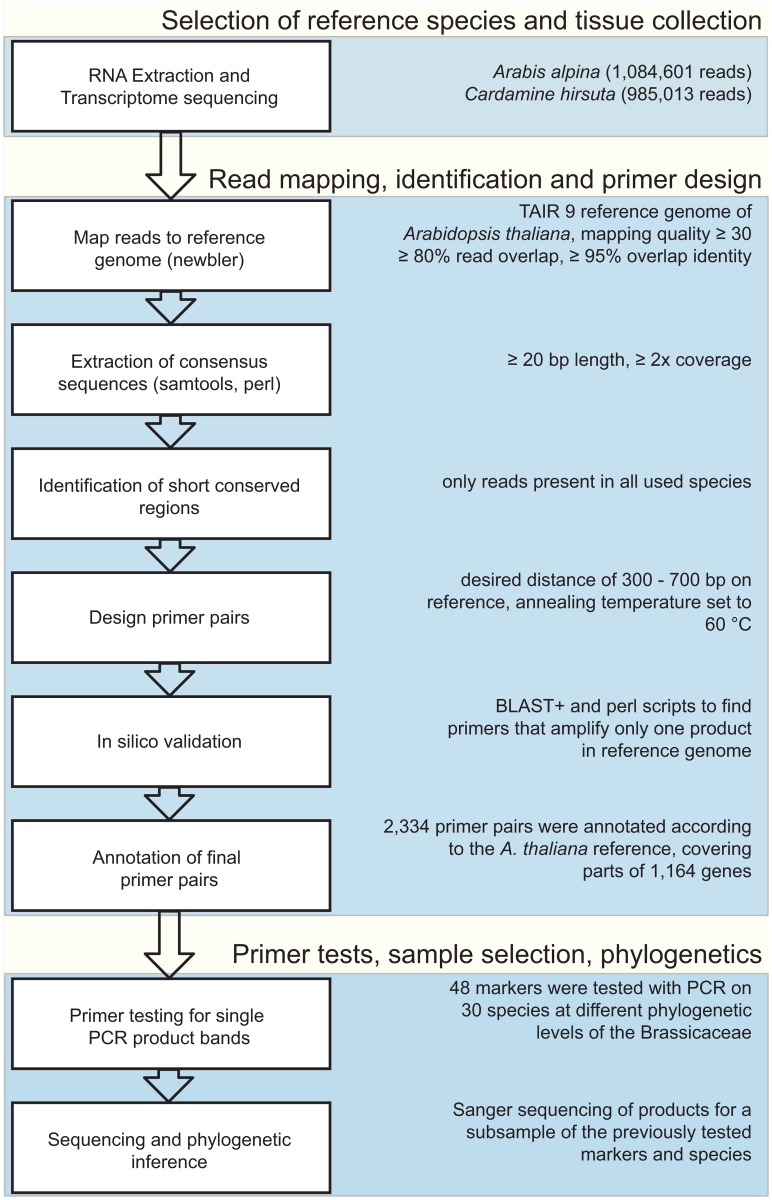
Workflow for the identification of conserved nucleotide sequences in multiple Brassicaceae species and subsequent primer design. Blue boxes refer to the three major steps in the workflow, white boxes indicate the general steps taken Explanations on the right provide specific results from this study.

To detect conserved sequence regions within the Brassicaceae, we used the available high- quality reference genome of *Arabidopsis thaliana* and additionally sequenced the transcriptomes of two divergent Brassicaceae species, *Cardamine hirsuta* and *Arabis alpina*. Sequencing reads from these two species were then aligned against the *A*. *thaliana* reference genome. These three species represent different lineages of the Brassicaceae family and hence should allow to detect shared, conserved regions suitable for designing universally applicable Brassicaceae primers. *Cardamine hirsuta* as well as *A*. *thaliana* are members of lineage I according to Al-Shehbaz [38], and diverged relatively early. *Arabis alpina* belongs to the Arabideae, the largest tribe of Brassicaceae [38], which is part of the expanded lineage II sensu Franzke *et al*. [39]. Dating the evolutionary history of Brassicaceae is particularly difficult, mostly due to few fossil records [40] and rapid radiation events [37,39,41]. A recent study [42] dated the split of lineage I and II to 27 million years ago (mya). Divergence time between *Cardamine* and *Arabidopsis* has been estimated to be at least 13 mya [35,43]. Therefore, the selection of these three different taxa reflects a divergence of nearly 30 million years and hence they cover a broad evolutionary range across the Brassicaceae, which allowed us to identify conserved regions among these species. The workflow is presented in Fig 1 and explained in detail below.

### Plant material, RNA extraction, normalization and sequencing

To gather as much of the exome as possible, 24 individuals from six populations of *A*. *alpina* and 28 individuals from nine populations of *C*. *hirsuta* were collected at different localities in Switzerland, to which we applied four growth chamber (Kälte 3000, Switzerland) stress treatments (S1 Table). "Drought" treated plants were harvested after seven days of water deprivation; "cold1" treated plants were exposed to 4°C for 24h, and "cold2" treated plants for two days to -6°C, while "heat" treatment involved two exposures to 40°C for 2h on two consecutive days. Since the localities of sampling are not nature reserves, and collected plants are neither protected nor endangered in these regions, we did not require sampling permits. This applies to all plant material used in our study.

mRNA from each species and treatment was extracted with RNeasy Plant Mini Kit (Qiagen, Netherlands) separately, and diluted to 300 ng/μl before they were equimolar pooled. Pooled mRNA was then reverse-transcribed with Super SMART PCR cDNA synthesis kit (Clontech, Takara Bio Europe SAS, France) in combination with Super Script III Reverse Transcriptase (Life Technologies, Invitrogen, USA). cDNA was amplified with iProof High-Fidelity DNA Polymerase (BioRad, USA) following the Super SMART protocol, normalized with TRIMMER (Evrogen, Russia) and sequenced on a Genome Sequencer FLX (GS-FLX; Roche, Switzerland) at the Functional Genomics Center Zurich (FGCZ; Zurich, Switzerland). Library synthesis was done with the GS-FLX Titanium Rapid Library Preparation Kit, and sequencing was then performed according to the Roche GS-FLX XLR70 Titanium emPCR and sequencing manuals. Each sample was sequenced twice on half a picotiter plate.

### Read mapping, identification, primer design

The GS-FLX raw sequencing reads (available from the NCBI sequence read archive, accession numbers SAMN03014707 and SAMN03014708) were extracted with the sff_extract tool and quality-controlled using custom-made Perl scripts. All raw read files had acceptable quality (average phred quality score above 20 to at least read position 350) and read length distributions (few short reads, high peak towards the long read lengths), and were thus included in the analysis. Reads were mapped onto the *A*. *thaliana* chromosomes [44] with the runMapping tool included in the Roche 454 software suite. Sequence alignments for *C*. *hirsuta* and *A*. *alpina* are available at the Dryad repository doi:10.5061/dryad.63j2j. Default mapping parameters were used except for minimum overlap length (set to 80%) and minimum overlap identity (set to 95%). The mapping output (ACE file) was converted to SAM format using the tools toAmos, bank-transact, and bank2contig from AMOS [45].

Using samtools [46] and custom-made Perl scripts, we then extracted start/end position information and nucleotide sequences for mapped regions at least 20 bp long and with at least 2x coverage per species. We decided for a low coverage to make our approach also suitable for smaller datasets. The mappings for *A*. *alpina* and *C*. *hirsuta* were then compared with Perl scripts, sequences which mapped to the same locations in *A*. *thaliana* and were conserved in all three species were extracted. These sequences were subsequently filtered for sequence pairs that had a minimal distance of 300 bp and a maximal distance of 700 bp on the *A*. *thaliana* reference genome, to adjust the length to current NGS sequencing platforms. These sequences were then used as input for primer3 v2.2.3 [47], to design forward and reverse PCR primers with lengths of 18 to 27 bp and annealing temperatures between 58 and 62°C. The following primer3 options were changed from default: PRIMER_MIN_SIZE = 18, PRIMER_OPT_SIZE = 20, PRIMER_MAX_SIZE = 27, PRIMER_MIN_TM = 58.0, PRIMER_OPT_TM = 60, PRIMER_MAX_TM = 62.0.

Our primer pairs were not *a priori* targeting exonic or intronic regions but instead targeted conserved regions across the three study species suitable for primer design and sufficiently close for PCR amplification and subsequent sequencing. Primer pairs were then tested *in silico* for uniqueness on the *A*. *thaliana* genome, using BLAST+ v2.2.23 [48] and custom-made Perl scripts using the following conditions: Primers were allowed to map to the genome with at most two nucleotide mismatches, and the maximal potential fragment size was set at 3,500 bp. Primer pairs potentially producing more than one fragment were discarded. Remaining primer pairs were annotated with the *A*. *thaliana* annotation from TAIR (http://arabidopsis.org).

A gene ontology (GO) analysis was performed to infer the biological functions of the annotated genes using DAVID [49,50] with false discovery rate (FDR) set to ≤ 0.05.

### Primer tests and sample selection

A subset of the primer pairs was then tested for amplification success and phylogenetic resolution in 30 Brassicaceae species covering the three major "lineages" [38].

We used herbarium specimens (collection Matthias Baltisberger, ETH Zurich) complemented by freshly collected and silica-gel dried leaves of plants from the University of Zurich Botanical Garden (Table 1).

**Table 1:** Species and samples used in this study for primer testing and sequencing.

Accession Name	Species	Herbarium Number	Collector and Date	Origin	PCR	Sequencing	Family	Lineage	Genus
-	*Aethionema saxatile* (L.) R.Br.	CH0Z-20100490	Steiger P., *et al*. 2010	Switzerland, San Salvatore TI 420 m asl	☑	☑	outgroup	☐	☐
-	*Arabidopsis halleri* (L.) O'Kane et Al-Shehbaz	-	Fischer M., 2011	45.90919° N 9.39207°	☑	☐	☐	☐	☐
Col-0	*Arabidopsis thaliana (*L.) Heynh. in Holl. & Heyn.	-	-	-	☑	☑	☑	☑	☐
-	*Arabis alpina* L.	Z/ZT MB 14820	Baltisberger M., 2011	Davos, Switzerland, 2200–2400 m asl	☑	☑	☑	☐	☑
-	*Arabis bellidifolia* Crantz s.l.	Z/ZT MB 14821	Baltisberger M., 2011	Davos, Switzerland, 2200–2400 m asl	☑	☑	☐	☐	☑
-	*Arabis ciliata* Clairv.	-	Gugerli F.	-	☑	☑	☐	☐	☑
-	*Arabis caerulea* All.	Z/ZT MB 14816	Baltisberger M., 2011	Davos, Switzerland, 2200–2400 m asl	☑	☑	☐	☐	☑
-	*Arabis subcoriacea* Gren.	Z/ZT MB 14814	Baltisberger M., 2011	Davos, Switzerland, 2200–2400 m asl	☑	☑	☐	☐	☑
-	*Barbarea vulgaris* R. Br.	XX0Z-19820365	Käser U., 2010	Botanical Garden Jaen, France	☑	☑	☐	☑	☐
-	*Biscutella laevigata* L.	Z/ZT MB 14815	Baltisberger M., 2011	Davos, Switzerland, 2200–2400 m asl	☑	☑	☑	outgroup	☐
-	*Boechera holboellii* (Hornem.) Á.Löve & D.Löve	-	-	-	☑	☑	☐	☑	☐
-	*Brassica nigra* (L.) W. D. J. Koch	XX0Z-20010028	Käser U., 2010	Botanical Gardens University Bonn-Germany	☑	☑	☑	☐	☐
-	*Braya humilis* (C. A. Meyer) B. L. Robinson	-	Marhold K.	Russia	☑	☑	☑	☐	☐
-	*Cardamine alpina* Willd.	Z/ZT MB 14836	Baltisberger M., 2011	Davos, Switzerland, 2200–2400 m asl	☑	☐	☐	☐	☐
-	*Cardamine amara* L. s.str.	Z/ZT MB 14813	Baltisberger M., 2011	Davos, Switzerland, 2100 m asl	☑	☐	☐	☐	☐
HAY1	*Cardamine hirsuta* L.	-	Shimizu-Inatsugi R.,	University of Zurich, Switzerland	☑	☑	☑	☑	☐
-	*Cardamine resedifolia* L.	Z/ZT MB 14818	Baltisberger M., 2011	Davos, Switzerland, 2200–2400 m asl	☑	☐	☐	☐	☐
-	*Cochlearia officinalis* L.	XX0Z-20001358	Schneeberger E.	Denmark, Bornholm, Teglkas, Shore	☑	☑	☑	☐	☐
-	*Diplotaxis tenuifolia* (L.) DC.	XX0Z-20000361	Käser U., 2009	Giardino Botanico Alpino Rezia-Bormio	☑	☑	☑	☐	☐
-	*Draba aizoides* L.	Z/ZT MB 14830	Baltisberger M., 2011	Davos, Switzerland, 2200–2400 m asl	☑	☑	☐	☐	outgroup
-	*Erysimum rhaeticum* (Hornem.) DC.	XX0Z-19770612	Käser U., 2009	Botanical Garden St. Gallen-Switzerland	☑	☐	☐	☐	☐
-	*Hesperis matronalis* L.	Z/ZT MB 14807	Baltisberger M., 2011	Davos, Switzerland, 1600 m asl	☑	☑	☑	☐	☐
-	*Hornungia alpina* (Siev.) O.Appel	Z/ZT MB 14817	Baltisberger M., 2011	Davos, Switzerland, 2300 m asl	☑	☐	☐	☐	☐
-	*Hornungia alpina* subsp. *brevicaulis* (Hoppe) O.Appel	Z/ZT MB 14834	Baltisberger M., 2011	Davos, Switzerland, 2200–2400 m asl	☑	☑	☑	☐	☐
-	*Iberis amara* L.	XX0Z-20100109	Käser U., 2010	EX BG Kiel; University Konstanz-Germany	☑	☑	☑	☐	☐
-	*Kernera saxatilis* (L.) Sweet	Z/ZT MB 14819	Baltisberger M., 2011	Davos, Switzerland, 2200–2400 m asl	☑	☑	☑	☐	☐
-	*Lepidium campestre* (L.) R. Br.	XX0Z-19963427	Käser U., 2010	-	☑	☑	☑	☑	☐
-	*Matthiola valesiaca* Boiss.	CH0Z-20060845	Affeltranger K., 2006	Switzerland, Binn VS 1280 m asl	☑	☑	☑	☐	☐
-	*Rorippa pyrenaica* (All.) Rchb.	-	Shimizu-Inatsugi R., 2007	Botanic Garden Zurich, Switzerland	☑	☑	☐	☑	☐
-	*Thlaspi ochroleucum* Boiss. & Heldr.	Z/ZT MB 14807	Baltisberger M., 2011	Switzerland	☑	☑	☑	☐	☐

The use of each species is divided into PCR, sequencing, family, "lineage" and genus. PCR indicates use for PCR amplification and sequencing indicates sequencing of the species, respectively. Family, "lineage" and genus refer to the application of species sequences at the three taxonomic levels that were phylogenetically tested in this study. The term "outgroup" refers to a taxon being sequenced and used as outgroup for phylogenetic analysis at a specific relationship level.

DNA was extracted with DNeasy Mini Kit (Qiagen), quantified with NanoDrop (ThermoFisher Scientific, USA) and Qubit (Invitrogen), and diluted to 50 ng/μl. Of the 2,334 identified primer pairs, 48 were selected (S6 Table) with a balanced number of EPIC and NPCL regions and even distribution across the reference genome.

PCR reactions consisted of 6.5 μl dH_2_O, 3 μl GoTaq buffer (Promega, USA), 1.5 μl MgCl_2_ (25mM), 1.5 μl dNTPs (2.5 mM), 0.75 μl forward primer, 0.75 μl reverse primer, 0.075 μl GoTaq (Promega), and 1 μl DNA (50 ng/μl). PCR conditions were designed to allow both Sanger sequencing and tagging for next-generation sequencing (S2 Table). Conditions for primer pairs amplified here were 94°C for 3 min, followed by 32 cycles of 94°C for 30 s, 58°C for 30 s, 72°C for 30 s, and 72°C for 7 min. PCRs were performed on Labcycler Basic (Sensoquest, Germany) and GeneAmp PCR System 9700 (Life Technologies, Invitrogen, USA). PCR amplifications were checked on 1% agarose gels in 1x TBE buffer. Single bands were counted as successful amplifications, whereas double bands, complex banding patterns or lack of amplification products were counted as failed amplifications.

We tested the newly designed primer pairs at different levels of phylogenetic relationships (i.e. family, "lineage" and genus). Overall, we amplified 13 nuclear marker regions (S6 Table) to reconstruct phylogenetic relationships among selected members of the Brassicaceae family.

The target species and primers are summarized in Table 1 and S6 Table. First, for the family-wide phylogeny, we chose 15 species that represent the depth of the mustard family: species from three "lineages" (I, II, and III) accepted in Brassicaceae, several species that are not assigned to "lineages" and basal taxa *sensu* Al-Shehbaz [38]. Six NPCLs with low sequence divergence between the mapping species and two EPIC markers containing two short introns with more polymorphic sites were used. Second, we selected “lineage I” of Brassicaceae *sensu* Al-Shehbaz [38] for our analysis at the "lineage" level using seven species, one NPCL region, two EPIC markers with a single intron, and three EPIC markers with two introns. Here, we focused on sequencing success and divergence of different types of loci. Third, *Arabis* was selected for the evaluation of PCR success and phylogeny reconstruction at the genus level, using five *Arabis* species and *Draba aizoides* as outgroup, and four primer pairs (one NPCL, two EPIC markers with two introns, one EPIC marker containing three introns).

Successfully amplified PCR products were purified using Exonuclease I and Fastap (Thermo Scientific) at 37°C for 45 min, followed by enzyme inactivation at 80°C for 15 min. Sanger sequencing was performed in 10 μl reaction volumes using 1 μl purified PCR product, 0.5 μl BigDye v3.1 (Applied Biosystems, USA), 1.9 μl sequencing buffer (5x concentration), 5.6 μl ddH_2_O and 1 μl of the sequencing primer (10 μM). Cycle sequencing reactions were performed with the following conditions: 60 s at 96°C followed by 35 cycles of 10 s at 95°C, 5 s at 50°C, and 4 min at 60°C. Reactions were cleaned using the BigDye Xterminator Purification kit according to manufacturer protocol (Applied Biosystems). Samples were analyzed on an ABI 3130xl DNA Analyser (Applied Biosystems).

### Phylogenetic analyses

We used Geneious v7.0.4. (Biomatters Ltd.) for quality check, trimming and sequence analysis. Sequence data are available in GenBank (accession numbers KM403211-KM403369). Alignments were created with mafft v7.0.17b [51], with the alignment strategy set to auto in the case of NPCL regions and single-intron covering EPIC markers, whereas E-INS-i, an iterative refinement method [51], was selected for EPIC markers with more than one intron. Alignments were manually inspected and adjusted if necessary. Stretches of monomeric repeats of over 8-bp length, microsatellite regions and ambiguous intron alignments were removed before analysis. Heterozygous sites were treated as ambiguities following IUPAC-IUB [52]. Successfully sequenced fragments were blasted in order to confirm the amplification of correct regions.

Aligned sequences were concatenated and analyzed using Sequence Matrix v1.7.8 [53] and are available at the Dryad repository (doi:10.5061/dryad.63j2j). Phylogenetic trees were calculated in Geneious v7.0.4. using the implemented RAxML v7.2.8. [54] for maximum likelihood analyses, and the MrBayes 3.2.1. [55] tool for Bayesian inference.

All concatenated alignments were calculated with partitions and substitution models according to the single markers. Family, "lineage" and genus approaches were run with the following setup for maximum likelihood analyses: For RAxML we used the favoured GTR substitution model by the jModelTest [56,57] AIC criterion. 1,000 bootstrap replicates with the setting "fast bootstrap calculation with detection of best ML tree" were performed to calculate bootstrap support (BS). The MrBayes substitution model to obtain posterior probabilities (PP) was also selected via AIC criterion in jModeltest. We ran 10,000,000 generations with random seed, a burn-in of 500,000 generations and 4 MCMC chains, three heated and one cold chain. Heated chain temperature was set to the default value of 0.2. Subsampling frequency in all MrBayes runs was set to 2,000, and outgroups were selected before analysis.

We compared the markers amplified by our primer pairs with previously used loci in terms of nucleotide diversity and parsimony-informative characters (PIC), which were assessed via MEGA5 [58]. Nucleotide diversity was calculated using "Mean Diversity in Entire Population" and PIC content was extracted from the "Sequence Data Explorer". GenBank accessions (S5 Table) of the nrDNA marker ITS, and the cpDNA regions *mat*K and *ndh*F of species from genera also used in our study were retrieved and aligned as described above.

## Results

### Read mapping, identification, primer design

A total of 1,084,601 and 985,013 sequencing reads were generated for *A*. *alpina* and *C*. *hirsuta*, respectively. 524,015 (48.3%) of the *A*. *alpina* reads mapped to *A*. *thaliana* and 488,249 (93.2%) had a mapping quality above 30, for *C*. *hirsuta*, respective numbers were 578,373 (58.7%) and 553,846 (95.8%) reads. Read length was between 40 and 1,188 nucleotides (median = 352, mean = 329).

We designed 2,334 primer pairs in 1,164 genes from short conserved anchor regions that are shared between *A*. *thaliana*, *C*. *hirsuta* and *A*. *alpina* (S6 Table). The mean length of these loci, compared to *A*. *thaliana*, was 535 bp (range: 339–787 bp) and their concatenated length equalled 1.23 Mbp.

The GO-term analysis revealed that genes amplified with our primer pairs cover many different pathways. Among the most overrepresented GO-terms were response to metal ions, response to abiotic stimuli, photosynthesis and carbohydrate biosynthesis (S3 Table).

### Primer tests and sample selection

PCR amplification success for 48 selected primer pairs in 30 members of the Brassicaceae family was on average 79.4% and varied between 50% and 100% for the different primer pairs, and between 35% and 100% depending on the species. We observed a trend that more diverged species had lower amplification success. In six cases, corresponding to 0.4% of all 1,440 PCRs performed, we detected two amplification products (S4 Table), which were counted as failed amplifications. The specificity of the primer pairs in successfully sequenced products was 100%, all 236 amplicons did match the targeted genes in a BLAST search.

### Phylogenetic analysis

**Fig 2:**
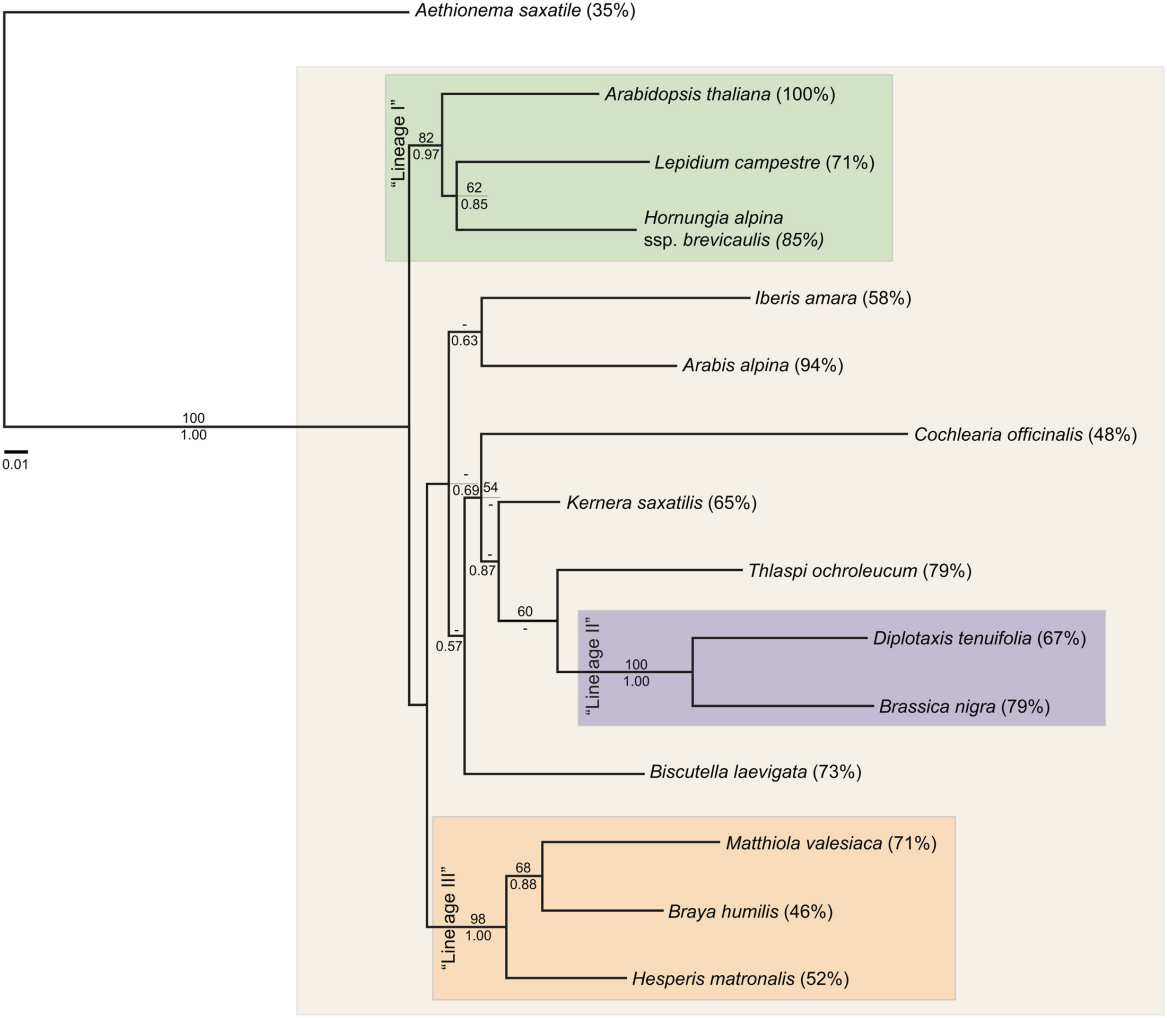
Phylogenetic inference at the family level. Best Maximum Likelihood phylogram of concatenated gene sequences are shown. Bootstrap support values and posterior probabilities are given above or below the corresponding branches, respectively. Values below 50/0.5 are omitted. "Lineage"-brackets refer to lineages *sensu* Al-Shehbaz (2012). Percentage amplification success per species is given in brackets next to each species name.

The phylogenetic tree at the family level based on eight concatenated nuclear markers (3,154 bp in total) showed good support for the three major Brassicaceae lineages (Fig 2). "Lineages I", "II" and "III" were strongly supported (BS ≥ 81; PP ≥ 0.98), but basal nodes of "lineage II" were not well resolved with either maximum likelihood or posterior probability inferences. The obtained phylogeny was compared with Couvreur *et al*. [35] and with the BrassiBase phylogeny [59]. Overall, we obtained good support for all branches identified in these studies. Incongruences were found in the placement of *Cochlearia*, as well as the non-basal position of *Biscutella* in our results, whereas traditionally the corresponding tribe (*Biscutelleae*) was placed as a sister to all major lineages.

**Fig 3:**
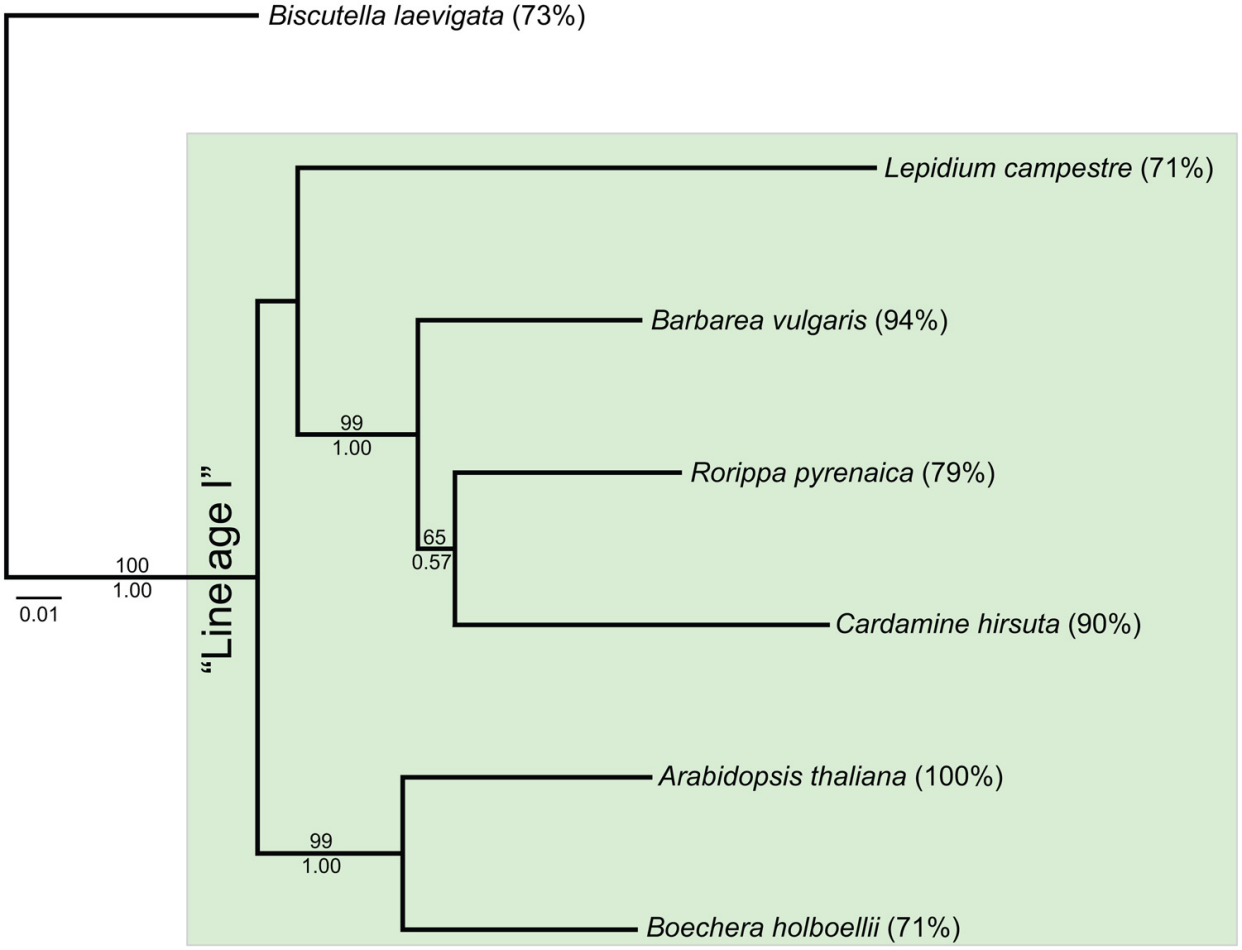
Phylogenetic inference at the "lineage" level. Best Maximum Likelihood phylogram of concatenated gene sequences are shown. Bootstrap support values and posterior probabilities are given above or below the corresponding branches, respectively. Values below 50/0.5 are omitted. Percentage amplification success per species is given in brackets next to each species name.

The concatenated dataset for phylogenetic analysis at the "lineage" level was 2,407 bp in length. *Barbarea vulgaris*, *C*. *hirsuta* and *Rorippa pyrenaica* formed one well-supported clade, (Fig 3; BS ≥ 99; PP = 1.00), *Boechera holboelli* and *A*. *thaliana* form another (Fig 3; BS ≥ 99; PP = 1.00).

**Fig 4:**
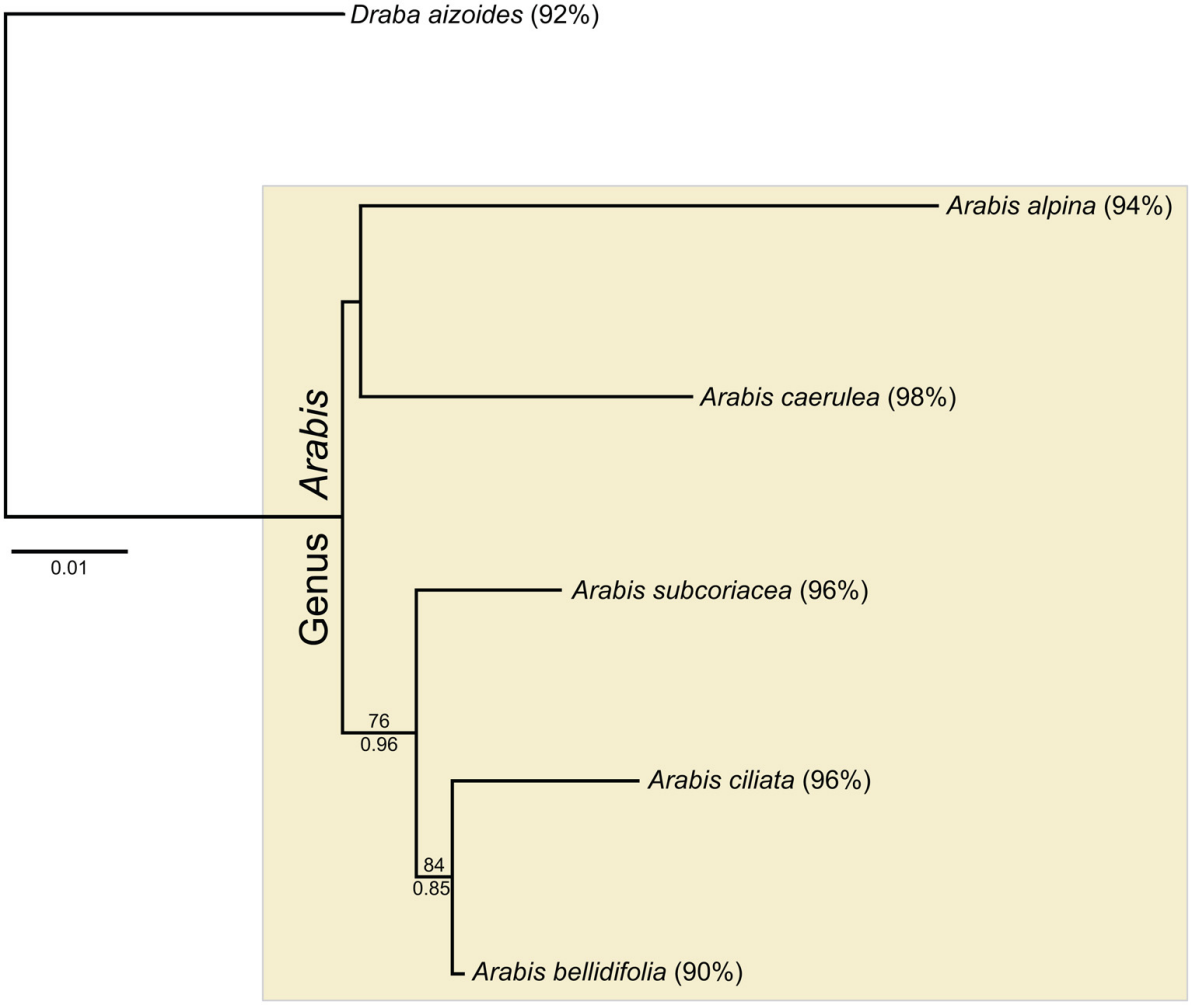
Phylogenetic inference at the genus level. Best Maximum Likelihood phylogram of concatenated gene sequences are shown. B Bootstrap support values and posterior probabilities are given above or below the corresponding branches, respectively. Values below 50/0.5 are omitted. Percentage amplification success per species is given in brackets next to each species name.

At the genus level, highly variable EPIC markers with multiple introns, as well as conserved regions, were sequenced. Most of the intron alignments had to be removed from the final alignment because of ambiguities. *Arabis subcoriacea*, *A*. *ciliata* and *A*. *bellidifolia* were grouped together with high support (BS = 76, PP = 0.96; Fig 4).

A comparison between markers used in this study and commonly applied markers ITS, *ndh*F and *mat*K at all three phylogenetic levels based on alignments revealed high variation in nucleotide diversity and PIC content of our markers. These values were always ranging between the levels of the compared ITS and plastid markers (Table 2).

**Table 2:** Details on alignments and markers used in our study as well as often-used markers at three taxonomic levels.

Family	*Bra254*	*Bra264*	*Bra637*	*Bra813*	*Bra1258*	*Bra1511*	*Bra1693*	*Bra2187*	ITS	*mat*K	*ndh*F
**Marker structure**	e	e	e	ii	e	e	e	ii	nrDNA	cpDNA	cpDNA
**No. of sequences**	14	15	13	13	14	14	12	13	14	14	12
**Sequencing success**	93.30%	100%	86.70%	86.70%	93.30%	93.30%	80.00%	86.70%	-	-	-
**Alignment length (bp)**	398	471	401	326	427	430	399	296	599	707	725
**PIC**	78	71	47	29	69	60	95	31	156	57	58
**PIC %**	19.60%	15.10%	11.70%	8.70%	16.20%	14.00%	23.80%	10.50%	26.00%	8.10%	8.00%
**Nucleotide diversity**	0.113	0.054	0.069	0.079	0.089	0.056	0.147	0.071	0.154	0.067	0.071
**GC content (%)**	47.40%	47.20%	41.60%	43.90%	47.60%	48.50%	43.90%	45.00%	54.00%	30.70%	25.50%
**Outgroup**	*Biscutella*	*Aethionema*	*Biscutella*	*Aethionema*	*Biscutella*	*Biscutella*	*Aethionema*	*Aethionema*	-	-	-
**Substitution model**	GTR+G+I	GTR+G+I	GTR+G	GTR+G	GTR+G+I	GTR+G+I	GTR+G+I	GTR+G+I	-	-	-
**"Lineage I"**	***Bra264***	***Bra406***	***Bra813***	***Bra1709***	***Bra1933***	***Bra2187***	**ITS**	***mat*K**	***ndh*F**		
**Marker structure**	e	ii	ii	i	i	ii	nrDNA	cpDNA	cpDNA		
**No. of sequences**	7	7	7	6	7	6	7	7	7		
**Sequencing success**	100%	100%	100%	87.50%	100%	87.50%	-	-	-		
**Alignment length (bp)**	470	449	480	336	157	515	599	725	651		
**PIC**	33	33	39	17	4	31	72	14	12		
**PIC %**	7.00%	7.30%	8.10%	5.10%	2.60%	5.60%	12.00%	1.90%	1.80%		
**Nucleotide diversity**	0.058	0.097	0.084	0.071	0.042	0.077	0.167	0.043	0.041		
**GC content (%)**	47.00%	37.40%	36.70%	45.70%	44.40%	41.10%	55.60%	30.90%	25.70%		
**Outgroup**	*Biscutella*	*Biscutella*	*Biscutella*	*Biscutella*	*Biscutella*	*Biscutella*	-	-	-		
**Substitution model**	GTR+G+I	GTR+G	HKY+I (GTR+I)	GTR+I	F81 (GTR)	GTR+G	-	-	-		
**Genus**	***Bra264***	***Bra320***	***Bra813***	***Bra1210***	**ITS**						
**Marker structure**	e	iiii	ii	ii	nrDNA						
**No. of sequences**	6	5	6	4	6						
**Sequencing success**	100%	83.30%	100%	66.70%	-						
**Alignment length (bp)**	470	451	548	528	617						
**PIC**	13	13	7	1	36						
**PIC %**	2.80%	2.90%	1.30%	0.20%	5.90%						
**Nucleotide diversity**	0.041	0.069	0.033	0.054	0.099						
**GC content (%)**	48.80%	38.60%	36.00%	44.20%	52.70%						
**Outgroup**	*Draba*	*Draba*	*Draba*	*Draba*	-						
**Substitution model**	GTR+G	GTR+I	GTR+G	GTR+G	-						

Marker structure refers to structure of amplified fragment (e = exon, i = one intron in the fragment, ii = two introns in the fragment, iii = three introns in the fragment). Sequencing success is the percentage of obtained, readable sequences. PIC refers to number of parsimony-informative characters in an alignment. PIC % shows the percentage of parsimony-informative sites within alignments. Substitution model refers to the applied substitution model for phylogenetic inference; values in brackets refer to alternative substitution model used in RAxML. Asterisk indicates that information is based on TAIR10.

## Discussion

The combination of high-throughput transcriptome sequencing in two distantly related Brassicaceae species and bioinformatics analysis in combination with the high quality *A*. *thaliana* reference genome allowed us to identify 2,334 primer pairs for nuclear markers located in 1,164 different genic regions. A GO overrepresentation analysis (S3 Table) revealed that responses to abiotic factors, such as responses to ions, were among the most overrepresented terms among these genes. The validation of a subset of primer pairs revealed that they can successfully be used for phylogenetic analyses at different taxonomic levels across the highly diverse mustard family. Other valuable features of the here provided new markers aside from their number are i) the possibility to choose levels of variability in amplified regions (S6 Table), and ii) to generate extensive sequence information for detailed analysis (up to 1.23 Mb length with all markers developed in this study). Thus, the published list of primer pairs may be of great value to studies of ecological genetics, adaptive trait evolution and population genetics in Brassicaceae.

Most previous studies that developed primer pairs amplifying across multiple species (cross-amplifying) focussed on specific markers, such as EPICs [26,28,29,31], nuclear protein coding loci NPCLs [25,27,30] or 3'UTR-anchored primers [25,29], depending on the scope of their scientific interest (Table 3). Among our 2,334 primer pairs are numerous EPIC, NPCL and UTR-anchored markers that were jointly identified in a single workflow, which greatly enhances the utility of our method and the supplied primer list (S6 Table). Because of the wide range of nucleotide variation that can be detected with different markers, partly dependent on their amplification of coding versus con-coding regions, they can be used for phylogenetic analyses at various taxonomic levels, but also for phylogeographic and population genetic studies [21].

**Table 3:** Comparison of our approach and other publications with similar scopes mentioned in our study.

Study	Taxon range	Method	Target loci	No. of loci found	Standard PCR conditions	Length (bp)
**Li *et al*. (2007)**	Order (Arctinopterygii)	database mining	NPCL	154	no	> 800
**Townsend *et al*. (2008)**	Order (Squamata)	database mining	NPCL	85	no	≥ 700
**Chenuil *et al*. (2010)**	Subkingdom (Eumetazoa)	database mining	EPIC	52	no	n. A.
**Li *et al*. (2010)**	Infraclass (Teleostei)	database mining	EPIC	210	yes	207–324
**Curto *et al*. (2012)**	Family (Lamiaceae)	database mining	EPIC	50	no	362–1717
**Shen *et al*. (2013)**	Subphylum (Vertebrata)	database mining	NPCL	102	yes (nested PCR)	510–1650
**Salas-Leiva *et al*. (2014)**	Order (Cycadales)	database mining	EPIC & UTR	46	no	259–1890
**Tonnabel *et al*. (2014)**	Genus (*Leucadendron*)	database mining & RNAseq	NPCL & UTR	7	no	277–796
**This study**	Family (Brassicaceae)	database mining & RNAseq	NPCL & EPIC & UTR	2,334	yes	339–787

Target loci indicate which fragments were targeted, standard PCR conditions indicates the availability of a uniform PCR protocol for all markers, length refers to fragment length of the regions found in a study. No. of loci found refers to the number of detected primer pairs or loci in the respective study.

A key aspect for the widespread use of the new primer pairs is their cross-species applicability. Our extensive tests revealed a PCR amplification success of 79.4% across a broad taxonomic range, and 99.6% of the amplified products showed single PCR bands, thus confirming the high specificity of the developed primers beyond the three initially sequenced taxa. As expected, amplification success varied between 100% in *A*. *thaliana*, which was used as a reference species, and 35% in *Aethionema saxatile*, which is only sister to the core Brassicaceae. Across closely related taxa to the study species, a very high number of successful PCR reactions was obtained. *Arabidopsis halleri* provided well-defined single bands in 96% of the tested primer pairs; the respective rates were 90–98% (mean 94%) for four included members of *Cardamine* and 90–98% (mean 94.8%) for the five tested species of *Arabis*. Overall, a limited taxon sampling can thus be sufficient to identify large numbers of conserved primer pairs suitable for studying a wide diversity of species. Moreover, the applicability of these primer pairs is not restricted to the lineages represented in the study, but may extend significantly beyond the studied species, as shown in Fig 2.

Lower amplification success for primer pairs in taxa that are phylogenetically distant to the studied species has also been reported by other studies, with success rates of 10.7% in the Lamiaceae [26] or 8.6% in Cycadales [29]. In our study, 10.4% of the tested primer pairs could be amplified in all 29 tested core Brassicaceae, and 6.25% in all 30 Brassicaceae. These percentages, if extended to the whole developed set of primer pairs would result in 244 primer pairs amplifying single PCR products in all core Brassicaceae or 147 primer pairs in all Brassicaceae.

Of further importance for the use of primers is that they amplify PCR products of suitable length for sequencing. While many studies report primers that amplify products > 1 kb long, we focused on relatively short amplification products in *A*. *thaliana*. This species has a much smaller genome than many other members of the Brassicaceae, amplicon sizes, especially for regions including introns, may thus often be undersized estimates. Nevertheless, many of our relatively short PCR products can be sequenced either in part or across their entire length not only with Sanger sequencing, but also with current NGS technologies, especially when using paired-end protocols.

Furthermore, many studies starting with a limited number of sequences tend to optimize PCR conditions separately for each marker. As a consequence, these markers often cannot be combined in multiplex assays that are most economic in combination with NGS technologies, so called target-enrichment strategies [60]. Our approach of keeping annealing temperatures in a narrow range for all primer pairs, together with the limited size range of PCR products, facilitates the joint analysis of multiple nuclear gene markers.

Direct Sanger sequencing of PCR products was successful with the primer pairs that were tested. Although the Sanger method does not allow to distinguish different alleles directly, which appear as heterozygous sites in electropherograms, we were still able to use this method to assess the utility of marker regions for phylogenetic inference. Resulting phylogenies at the family and "lineage" levels were largely congruent with previous results [35,59]. Phylogenetic resolution at the genus level was low, which may be the consequence of the low number of included species or low phylogenetic information content of two of the four marker regions used, potentially leading to an increase in noise levels [61].

Comparing markers from our study with commonly used gene regions showed that our primer pairs are able to amplify fragments with high and low potential sequence divergence and PIC content. The nucleotide diversity and PIC number of loci from this study were found to range, depending on the studied locus and relationship levels, between low diversity values similar to the compared plastid markers *mat*K and *ndh*F, and levels that are similar to the nrDNA marker ITS, which is a fast evolving region (Table 2). These considerable differences in sequence evolution underline previous statements that single-gene trees may often not reflect the true evolutionary history of a taxon [14,62], thus it is of utmost importance to include many unlinked loci in evolutionary analyses. Nowadays NGS technologies coupled with target-enrichment methods, which have been used e.g. in phylogenetic [63–65] and phylogeography studies [66,67], may help overcome the potential mismatches of gene and species-trees. Therefore, our method and primer list may be used for such target-enrichment approaches, and present useful tools to study a large number of conserved marker regions in a cost-effective and fast manner across a broad range of taxa in the Brassicaceae family.

Available annotations for our primer list can be used to focus on specific groups of genes in targeted evolutionary, ecological or genomic studies that focus, for example, on genetic diversity and evolution of stress-responsive genes.

Altogether, we anticipate that the set of 2,334 nuclear gene markers presented here will benefit the Brassicaceae research community and facilitate future analyses of phylogenetic relationships and evolutionary processes in this highly diverse plant group [39,68]. Essential information, such as amplicon type, length or gene ID based on the *A*. *thaliana* reference genome, are readily available (S6 Table) and hence ready to use for further studies. Finally, and most importantly, the large number of available nuclear gene markers will hopefully allow changing our perspective to move away from the analysis of a few genes that undergo uniparental inheritance or concerted evolution towards a truly genome-wide analysis of diversity and divergence.

## Supporting Information

S1 Table Plant accessions and treatments for transcriptome sequencing of *A*. *alpina* and *C*. *hirsuta*. Sample origin refers to sampling location, tissue type refers to sampled tissue for RNA extraction. Treatment refers to applied stress treatment with details in brackets, before tissue was collected. ^1^ Pooled samples from three populations from 8.86°E 47.06°N, 8.91°E 47.06°N and 9.05°E 47.09°N. ^2^ Pooled samples from three populations from 9.35°E 47.24°N, 9.02°E 47.08°N and 9.43°E 46.97°N.
(PDF)

S2 Table PCR protocol for Fluidigm Amplicon Tagging (http://www.fluidigm.com/access-array-system.html). Tag sequences: forward 5’-ACACTGACGACATGGTTCTACA-3’ and reverse 5’-TACGGTAGCAGAGACTTGGTCT-3’.
(PDF)

S3 Table GO-overrepresentation analysis of all 1,164 genes amplified (at least in part) with primer pairs developed in this study, sorted by false-discovery rate (FDR) values. Analysis was performed by the online tool DAVID 6.7.
(PDF)

S4 Table PCR amplification success of 48 primer pairs tested on 30 Brassicaceae species. Numbers 0,1 and 2 refer to the number of bands obtained on agarose gels after PCR. Amplification success per marker and amplification success per species were calculated by adding all successful single product amplifications and dividing it by the overall number of tested species or markers, respectively.
(PDF)

S5 Table Sequences retrieved from GenBank for comparisons between commonly used markers and markers amplified with primer pairs developed in this study. (PDF)

S6 Table 2,334 primer pairs developed in this study. (XLSX)
