# Supplementary material for: Efficient Detection of Novel Nuclear Markers for Brassicaceae by Transcriptome Sequencing
Source: PLoS One. 2015 Jun 10;10(6):e0128181. doi: 10.1371/journal.pone.0128181 (PMC4465667; doi:10.1371/journal.pone.0128181)
Supplement: S1 Table — Sample origin refers to sampling location, tissue type refers to sampled tissue for RNA extraction. Treatment refers to applied stress treatment with details in brackets, before tissue was collected. 1 Pooled samples from three populations from 8.86°E 47.06°N, 8.91°E 47.06°N and 9.05°E 47.09°N. 2 Pooled samples from three populations from 9.35°E 47.24°N, 9.02°E 47.08°N and 9.43°E 46.97°N. (PDF) [file pone.0128181.s001.pdf]

S1 Table. Plant accessions and treatments for transcriptome sequencing of Swiss *A. alpina* and *C. hirsuta*.*

Species	Sample Origin	Tissue type	Treatment	Sample
Arabis alpina	multiple[1]	leaf	Cold (1 day at 4°C)	6
Arabis alpina	multiple[1]	leaf	Cold (1 day at 4°C)	7
Arabis alpina	multiple[1]	root	Cold (1 day at 4°C)	11
Arabis alpina	multiple[1]	root	Cold (1 day at 4°C)	12
Arabis alpina	multiple[1]	root	Cold (1 day at 4°C)	13
Arabis alpina	multiple[1]	leaf	Drought	50
Arabis alpina	multiple[1]	root	Drought	58
Arabis alpina	multiple[1]	leaf	Drought	59
Arabis alpina	multiple[1]	leaf	Drought	63
Arabis alpina	multiple[1]	root	Drought	65
Arabis alpina	multiple[1]	leaf	Cold (2 days at -6°C)	118
Arabis alpina	multiple[1]	root	Cold (2 days at -6°C)	137
Arabis alpina	multiple[1]	root	Cold (2 days at -6°C)	140
Arabis alpina	multiple[1]	leaf	Heat (2 days at 40°C x2h)	159
Arabis alpina	multiple[1]	root	Heat (2 days at 40°C x2h)	199
Arabis alpina	multiple[1]	root	Heat (2 days at 40°C x2h)	200
Arabis alpina	multiple[1]	root	Heat (2 days at 40°C x2h)	202
Arabis alpina	multiple[2]	leaf	Cold (1 day at 4°C)	19
Arabis alpina	multiple[2]	root	Cold (1 day at 4°C)	23
Arabis alpina	multiple[2]	root	Cold (1 day at 4°C)	26
Arabis alpina	multiple[2]	leaf	Cold (2 days at -6°C)	129
Arabis alpina	multiple[2]	root	Cold (2 days at -6°C)	135
Arabis alpina	multiple[2]	leaf	Heat (2 days at 40°C x2h)	167
Arabis alpina	multiple[2]	root	Heat (2 days at 40°C x2h)	205
Arabis alpina	8.53°E 46.53°N	silique, flower	no	U076-2
Arabis alpina	8.53°E 46.53°N	flower	no	U719-2
Cardamine hirsuta	8.37°E 47.21°N	leaf	Cold (1 day at 4°C)	34
Cardamine hirsuta	8.37°E 47.21°N	root	Cold (1 day at 4°C)	38
Cardamine hirsuta	8.37°E 47.21°N	leaf	Drought	70
Cardamine hirsuta	8.37°E 47.21°N	root	Drought	71
Cardamine hirsuta	8.37°E 47.21°N	leaf	Cold (2 days at -6°C)	103
Cardamine hirsuta	8.37°E 47.21°N	root	Cold (2 days at -6°C)	142
Cardamine hirsuta	8.37°E 47.21°N	leaf	Heat (2 days at 40°C x2h)	171
Cardamine hirsuta	8.37°E 47.21°N	leaf	Heat (2 days at 40°C x2h)	173
Cardamine hirsuta	8.37°E 47.21°N	root	Heat (2 days at 40°C x2h)	191
Cardamine hirsuta	8.56°E 46.01°N	leaf	Cold (1 day at 4°C)	27
Cardamine hirsuta	8.56°E 46.01°N	root	Cold (1 day at 4°C)	30
Cardamine hirsuta	8.56°E 46.01°N	leaf	Drought	93
Cardamine hirsuta	8.56°E 46.01°N	root	Drought	95
Cardamine hirsuta	8.56°E 46.01°N	leaf	Cold (2 days at -6°C)	105
Cardamine hirsuta	8.56°E 46.01°N	root	Cold (2 days at -6°C)	154
Cardamine hirsuta	8.56°E 46.01°N	root	Cold (2 days at -6°C)	156
Cardamine hirsuta	8.56°E 46.01°N	leaf	Heat (2 days at 40°C x2h)	174
Cardamine hirsuta	8.56°E 46.01°N	root	Heat (2 days at 40°C x2h)	194
Cardamine hirsuta	6.03°E 46.11°N	leaf	Cold (1 day at 4°C)	39
Cardamine hirsuta	6.03°E 46.11°N	root	Cold (1 day at 4°C)	46
Cardamine hirsuta	6.03°E 46.11°N	root	Cold (1 day at 4°C)	48
Cardamine hirsuta	6.03°E 46.11°N	leaf	Drought	80
Cardamine hirsuta	6.03°E 46.11°N	root	Drought	86
Cardamine hirsuta	6.03°E 46.11°N	root	Drought	88
Cardamine hirsuta	6.03°E 46.11°N	leaf	Cold (2 days at -6°C)	113
Cardamine hirsuta	6.03°E 46.11°N	root	Cold (2 days at -6°C)	147
Cardamine hirsuta	6.03°E 46.11°N	leaf	Heat (2 days at 40°C x2h)	180
Cardamine hirsuta	6.03°E 46.11°N	root	Heat (2 days at 40°C x2h)	184
Cardamine hirsuta	8.43°E 47.16°N	flower	no	206
Cardamine hirsuta	8.43°E 47.16°N	flower	no	207
Cardamine hirsuta	8.43°E 47.16°N	flower	no	208
Cardamine hirsuta	8.43°E 47.16°N	flower	no	209
Cardamine hirsuta	8.43°E 47.16°N	flower	no	210

*Sample origin refers to sampling location, tissue type refers to sampled tissue for RNA extraction, Treatment refers to applied stress treatment with details in brackets,before tissue was collected, respectively. [1] Pooled samples from three populations from 8.86°E 47.06°N, 8.91°E 47.06°N and 9.05°E 47.09°N. [2] Pooled samples from three populations from 9.35°E 47.24°N, 9.02°E 47.08°N and 9.43°E 46.97°N.
