# Supplementary material for: Efficient Detection of Novel Nuclear Markers for Brassicaceae by Transcriptome Sequencing
Source: PLoS One. 2015 Jun 10;10(6):e0128181. doi: 10.1371/journal.pone.0128181 (PMC4465667; doi:10.1371/journal.pone.0128181)
Supplement: S2 Table — Tag sequences: forward 5’-ACACTGACGACATGGTTCTACA-3’ and reverse 5’-TACGGTAGCAGAGACTTGGTCT-3’. (PDF) [file pone.0128181.s002.pdf]

S2 Table. PCR protocol for Fluidigm Amplicon Tagging (http://www.fluidigm.com/access-array-system.html).*

Number of cycles	T (°C)	Time
	95	3 min
10 {	95	15 s
	60	30 s
	72	1 min
2 {	95	15 s
	80	30 s
	60	30 s
	72	1 min
8 {	95	15 s
	60	30 s
	72	1 min
2 {	95	15 s
	80	30 s
	60	30 s
	72	1 min
8 {	95	15 s
	60	30 s
	72	1 min
5 {	95	15 s
	80	30 s
	60	30 s
	72	1 min
	72	7 min
	4	∞

*Tag sequences: forward 5'-ACACTGACGACATGGTTCTACA-3' and reverse 5'-TACGGTAGCAGAGACTTGGTCT-3'.
