# Supplementary material for: Efficient Detection of Novel Nuclear Markers for Brassicaceae by Transcriptome Sequencing
Source: PLoS One. 2015 Jun 10;10(6):e0128181. doi: 10.1371/journal.pone.0128181 (PMC4465667; doi:10.1371/journal.pone.0128181)
Supplement: S3 Table — Analysis was performed by the online tool DAVID 6.7. (PDF) [file pone.0128181.s003.pdf]

S3 Table. GO-overrepresentation analysis* of all 1,164 genes amplified (at least in part) with primer pairs developed in this study, sorted by false-discovery rate (FDR) values.

GO-Term	No. of genes	FDR
response to cadmium ion	49	8.85E-07
response to metal ion	52	8.75E-06
nitrogen compound biosynthetic process	62	1.20E-05
response to abiotic stimulus	113	2.80E-05
hexose metabolic process	28	2.89E-04
carbohydrate biosynthetic process	37	3.24E-04
monosaccharide metabolic process	30	5.97E-04
cellular carbohydrate catabolic process	26	6.11E-04
response to inorganic substance	59	0.001531559
carboxylic acid biosynthetic process	48	0.002492413
organic acid biosynthetic process	48	0.002492413
cellular carbohydrate biosynthetic process	29	0.003030663
photosynthesis	27	0.006668279
cellular glucan metabolic process	22	0.006986778
alcohol catabolic process	21	0.008892257
response to salt stress	43	0.012155865
amine biosynthetic process	29	0.013688571
glucan metabolic process	24	0.015633436
cellular polysaccharide metabolic process	24	0.021765577
response to temperature stimulus	41	0.023934446
glucose catabolic process	19	0.028219854
hexose catabolic process	19	0.03226425
monosaccharide catabolic process	19	0.03226425
glucose metabolic process	20	0.032467233
response to osmotic stress	44	0.037063436
carbohydrate catabolic process	28	0.038535592

*Analysis was performed by the online tool DAVID 6.7 [1,2].

References

1. Huang DW, Sherman BT, Lempicki RA (2009) Systematic and integrative analysis of large gene lists using DAVID bioinformatics resources. Nature Protocols 4: 44-57.
2. Huang DW, Sherman BT, Zheng X, Yang J, Imamichi T, et al. (2009) Extracting biological meaning from large gene lists with DAVID. Current Protocols in Bioinformatics 13: Unit 13.11.
