# Supplementary material for: Efficient Detection of Novel Nuclear Markers for Brassicaceae by Transcriptome Sequencing
Source: PLoS One. 2015 Jun 10;10(6):e0128181. doi: 10.1371/journal.pone.0128181 (PMC4465667; doi:10.1371/journal.pone.0128181)
Supplement: S4 Table — Numbers 0,1 and 2 refer to the number of bands obtained on agarose gels after PCR. Amplification success per marker and amplification success per species were calculated by adding all successful single product amplifications and dividing it by the overall number of tested species or markers, respectively. (PDF) [file pone.0128181.s004.pdf]

S4 Table. PCR amplification success of 48 primer pairs tested on 30 Brassicaceae species.*

Marker	*Aethionema saxatile*	*Arabidopsis thaliana*	*Arabidopsis halleri*	*Arabis alpina*	*Arabis bellidifolia*	*Arabis caerulea*	*Arabis ciliata*	*Arabis subcoriacea*	*Barbarea vulgaris*	*Biscutella laevigata*	*Boechera holboellii*	*Brassica nigra*	*Braya humilis*	*Cardamine alpina*	*Cardamine amara*	*Cardamine hirsuta*	*Cardamine resedifolia*	*Cochlearia officinalis*	*Diplotaxis tenuifolia*	*Draba aizoides*	*Erysimum rhaeticum*	*Hesperis matronalis*	*Hornungia alpina*	*Hornungia alpina* ssp. *brevicaulis*	*Iberis amara*	*Kernera saxatilis*	*Lepidium campestre*	*Mathiola valesiaca*	*Thlaspi ochroleucum*	*Rorippa pyrenaica*	amplification success per marker
Bra13	1	1	1	0	1	1	1	1	1	0	1	1	0	1	1	1	1	1	1	1	1	0	1	1	0	0	1	1	1	1	**0.80**
Bra48	1	1	1	1	1	1	1	1	1	1	0	1	1	1	1	1	1	1	1	1	1	1	1	1	1	1	1	1	1	0	**0.93**
Bra162	0	1	1	1	1	1	1	1	1	1	1	1	1	1	1	1	1	0	0	0	1	0	0	0	1	1	0	1	1	1	**0.73**
Bra254	0	1	1	1	1	1	1	1	1	1	1	1	1	1	1	1	1	1	1	1	1	1	1	1	1	1	1	1	1	1	**0.97**
Bra262	0	1	0	1	1	1	1	1	1	1	1	1	0	1	1	0	1	0	1	1	1	0	0	1	1	1	0	0	1	1	**0.70**
Bra264	1	1	1	1	1	1	1	1	1	1	1	1	1	1	1	1	1	1	1	1	1	1	1	1	1	1	1	1	1	1	**1.00**
Bra272	0	1	1	1	1	1	1	0	0	0	0	1	0	0	0	0	0	0	0	1	1	1	1	1	1	0	0	1	0	1	**0.50**
Bra320	1	1	1	1	1	1	1	1	1	1	1	1	0	1	1	1	0	0	0	1	1	1	1	1	0	1	0	0	0	1	**0.73**
Bra344	0	1	1	1	1	1	1	1	1	1	1	1	1	1	1	0	1	1	1	1	1	0	1	0	1	1	1	1	1	0	**0.83**
Bra398	1	1	1	1	1	1	1	1	1	1	1	1	1	1	1	1	1	1	1	1	1	1	1	1	1	1	1	1	1	1	**1.00**
Bra406	0	1	1	1	1	1	1	1	1	1	1	0	1	1	1	1	1	0	0	1	1	0	0	0	0	1	1	1	0	1	**0.70**
Bra424	0	1	1	1	0	1	1	0	1	0	1	0	0	1	0	1	1	0	0	1	1	0	1	1	0	0	0	0	1	1	**0.53**
Bra453	0	1	1	1	1	1	1	1	0	0	0	1	0	1	1	1	1	1	0	1	0	0	1	1	0	1	1	1	0	0	**0.63**
Bra583	0	1	1	1	1	1	1	1	1	0	0	0	0	1	1	1	1	0	0	1	0	1	1	1	0	1	0	0	0	0	**0.57**
Bra605	1	1	1	1	1	1	1	1	1	1	1	1	1	1	1	1	1	1	1	1	1	1	1	1	1	0	1	1	1	1	**0.97**
Bra633	0	1	1	1	1	1	1	1	1	1	0	0	0	1	1	1	1	0	0	1	1	1	1	1	0	1	0	0	1	1	**0.70**
Bra637	0	1	1	1	1	1	1	1	1	1	1	1	0	1	1	1	1	1	1	1	1	1	0	1	1	1	1	1	1	1	**0.90**
Bra754	0	1	1	1	1	1	1	1	1	1	0	1	1	1	1	1	1	1	1	1	1	0	1	1	1	1	0	1	1	0	**0.83**
Bra758	0	1	1	1	1	1	1	1	1	1	0	0	0	1	1	1	1	0	1	1	0	0	1	1	1	1	1	0	1	1	**0.73**
Bra813	1	1	1	1	1	1	1	1	1	0	1	1	1	1	1	1	1	0	1	1	1	1	1	0	1	1	1	1	1	1	**0.90**
Bra978	1	1	1	2	1	1	1	1	1	1	1	1	0	1	1	1	1	0	0	1	1	1	1	1	0	0	1	0	1	1	**0.77**
Bra1148	1	1	1	1	0	1	1	1	1	1	0	0	1	1	1	1	1	0	1	1	1	0	0	0	1	1	1	1	1	1	**0.77**
Bra1149	0	1	1	1	1	1	1	1	1	1	0	1	0	1	1	1	1	0	0	1	1	0	1	0	0	1	0	0	0	1	**0.63**
Bra1150	1	1	1	1	1	1	1	1	1	1	1	1	0	1	1	1	1	0	1	1	1	0	1	1	1	1	1	1	1	1	**0.90**
Bra1210	0	1	0	1	0	1	1	1	1	1	1	1	1	1	1	1	1	1	0	1	1	1	1	1	1	0	0	1	1	0	**0.77**
Bra1253	0	1	1	1	1	1	1	1	1	2	0	0	0	1	1	1	1	0	1	1	1	1	1	1	1	0	1	1	1	1	**0.77**
Bra1258	0	1	1	1	1	1	1	1	1	1	1	1	1	1	1	1	1	1	1	1	1	1	1	1	1	1	1	0	1	1	**0.93**
Bra1327	0	1	1	1	1	1	1	1	1	2	1	1	0	1	1	1	1	0	0	1	1	0	0	1	0	0	1	0	1	1	**0.67**
Bra1402	0	1	1	1	1	1	1	1	1	1	1	1	0	1	1	1	1	1	1	1	1	0	1	1	0	1	0	1	1	1	**0.83**
Bra1482	0	1	1	1	1	1	1	1	1	1	1	1	0	1	1	1	1	0	1	0	1	0	1	1	0	1	1	0	1	1	**0.77**
Bra1490	1	1	1	1	1	1	1	1	1	1	1	1	1	1	1	1	1	0	1	1	1	0	0	0	1	0	1	1	1	1	**0.83**
Bra1497	1	1	1	1	1	1	1	1	1	1	1	1	0	1	1	0	1	1	1	1	1	1	1	1	0	1	1	1	1	1	**0.90**
Bra1511	0	1	1	1	1	1	1	1	1	1	1	1	1	1	1	1	1	1	1	1	1	1	1	1	1	1	1	1	1	1	**0.97**
Bra1584	0	1	1	1	1	1	1	1	2	1	0	1	1	1	1	1	1	1	0	0	1	1	1	1	0	1	0	1	0	2	**0.70**
Bra1617	0	1	1	1	1	1	1	1	1	1	1	1	1	1	1	1	0	1	1	1	1	1	1	1	1	1	1	1	1	1	**0.93**
Bra1637	0	1	1	1	1	1	1	1	1	0	1	0	0	1	1	1	1	0	0	1	1	0	1	1	0	0	1	0	1	1	**0.67**
Bra1693	1	1	1	1	1	1	1	1	1	1	0	1	0	1	0	1	1	0	1	1	1	1	1	1	1	1	1	1	0	1	**0.83**
Bra1709	0	1	1	1	0	1	1	1	1	1	1	1	0	1	1	1	1	0	0	1	1	0	1	1	0	0	1	1	0	0	**0.67**
Bra1811	1	1	1	1	1	1	1	1	1	1	1	1	1	1	1	1	1	1	1	1	1	0	1	1	1	0	1	1	1	0	**0.90**
Bra1933	2	1	1	1	1	1	1	1	1	0	1	1	1	1	1	1	1	0	1	1	1	1	1	1	1	1	1	1	0	1	**0.87**
Bra2076	0	1	1	1	1	1	1	1	1	1	1	1	1	1	1	1	1	1	1	1	1	1	1	1	0	0	1	1	1	1	**0.90**
Bra2161	1	1	1	1	1	1	1	1	1	0	0	0	0	1	1	1	1	0	1	1	1	0	1	1	1	1	1	0	1	1	**0.77**
Bra2173	0	1	1	1	1	1	1	1	1	1	1	1	0	1	1	1	1	1	1	1	1	0	1	1	0	1	1	1	1	1	**0.87**
Bra2187	1	1	1	1	1	1	1	1	1	1	1	1	0	1	1	1	1	1	1	1	0	1	1	1	1	0	1	1	1	1	**0.90**
Bra2220	0	1	1	1	1	1	0	1	1	0	1	0	0	1	1	1	1	0	0	1	1	0	1	1	0	0	0	0	1	0	**0.57**
Bra2221	1	1	1	1	1	1	1	1	1	1	1	1	1	1	1	1	1	1	1	1	1	1	1	1	1	1	1	1	1	1	**1.00**
Bra2223	0	1	1	0	0	1	1	1	1	1	1	1	1	1	1	1	1	1	1	1	1	1	1	1	1	0	1	1	1	1	**0.87**
Bra2257	0	1	1	1	1	0	0	1	1	0	0	1	0	1	1	0	1	0	1	0	0	0	1	1	0	0	0	1	1	1	**0.53**
**amplification success per species**	**0.35**	**1.00**	**0.96**	**0.94**	**0.90**	**0.98**	**0.96**	**0.96**	**0.94**	**0.73**	**0.71**	**0.79**	**0.46**	**0.98**	**0.94**	**0.90**	**0.94**	**0.48**	**0.67**	**0.92**	**0.90**	**0.52**	**0.85**	**0.85**	**0.58**	**0.65**	**0.71**	**0.71**	**0.79**	**0.79**	

*Numbers 0,1 and 2 refer to the number of bands obtained on agarose gels after PCR. Amplification success per marker and amplification success per species were calculated by adding all successful single product amplifications and dividing it by the overall number of tested species or markers, respectively.
