# Supplementary material for: Efficient Detection of Novel Nuclear Markers for Brassicaceae by Transcriptome Sequencing
Source: PLoS One. 2015 Jun 10;10(6):e0128181. doi: 10.1371/journal.pone.0128181 (PMC4465667; doi:10.1371/journal.pone.0128181)
Supplement: S5 Table — (PDF) [file pone.0128181.s005.pdf]

S5 Table. Sequences retrieved from GenBank for comparison between commonly used markers and markers amplified with primer pairs developed in this study.

Species*	GenBank ID*	Locus*	used for*	Species	GenBank ID	Locus	used for
Aethionema saxatile	GQ284853.1	ITS	family	*Cardamine hirsuta*	HM850748.1	matK	lineage
Aethionema saxatile	EU371817.1	matK	family	*Cardamine hirsuta*	HQ616529.1	ndhF	lineage
Aethionema saxatile	DQ288726.1	ndhF	family	*Cochlearia officinalis*	HQ268642.1	ITS	family
Arabidopsis thaliana	AJ232900.1	ITS	family	*Cochlearia officinalis*	JN895761.1	matK	family
Arabidopsis thaliana	DQ528813.1	ITS	lineage	*Cochlearia officinalis*	AY514390.1	ndhF	family
Arabidopsis thaliana	AF144378.1	matK	lineage	*Diplotaxis tenuifolia*	EF601913.1	ITS	family
Arabidopsis thaliana	AF144378.1	matK	family	*Diplotaxis tenuifolia*	HE967405.1	matK	family
Arabidopsis thaliana	JQ323085.1	ndhF	family	*Draba aizoides*	GU202434.1	ITS	genus
Arabidopsis thaliana	JQ323085.1	ndhF	lineage	*Erysimum perofskianum*	DQ406762.1	matK	lineage
Arabis alpina	AF137559.1	ITS	family	*Hesperis matronalis*	DQ357547.1	ITS	family
Arabis alpina	DQ060111.1	ITS	genus	*Hesperis matronalis*	HQ593319.1	matK	family
Arabis alpina	KC474054.1	matK	family	*Hesperis matronalis*	DQ288776.1	ndhF	family
Arabis caerulea	JQ919837.1	ITS	genus	*Hornungia alpina*	DQ310527.1	ITS	family
Arabis ciliata	FJ187950.1	ITS	genus	*Hornungia petraea*	JN893991.1	matK	family
Arabis jacquinii	AJ232919.1	ITS	genus	*Hornungia petraea*	KF023023.1	ndhF	family
Arabis pumila	KC412276.1	ITS	genus	*Iberis amara*	AJ440311.1	ITS	family
Barbarea vulgaris	AJ232915.2	ITS	lineage	*Iberis amara*	GQ424589.1	matK	family
Barbarea vulgaris	HQ593190.1	matK	lineage	*Iberis amara*	AY514391.1	ndhF	family
Barbarea vulgaris	AF064652.1	ndhF	lineage	*Kernera saxatilis*	AJ440313.1	ITS	family
Biscutella laevigata	DQ452056.1	ITS	family	*Lepidium campestre*	AF055197.1	ITS	family
Biscutella laevigata	KF022694.1	ITS	lineage	*Lepidium campestre*	AF055197.1	ITS	lineage
Biscutella laevigata	KF023014.1	ndhF	lineage	*Lepidium campestre*	HQ593342.1	matK	lineage
Biscutella neustriaca	JN987160.1	matK	family	*Lepidium campestre*	HQ593342.1	matK	family
Biscutella neustriaca	JN987160.1	matK	lineage	*Lepidium campestre*	JQ323087.1	ndhF	family
Boechera canadensis	DQ165348.1	ITS	lineage	*Lepidium campestre*	JQ323087.1	ndhF	lineage
Boechera drummondii	AF064658.1	ndhF	lineage	*Matthiola incana*	HM850753.1	matK	family
Brassica incana	KF023015.1	ndhF	family	*Matthiola incana*	KF023009.1	ndhF	family
Brassica nigra	AY722422.1	ITS	family	*Matthiola parviflora*	DQ357568.1	ITS	family
Brassica nigra	JN894593.1|	matK	family	*Rorippa gambelii*	AF198154.1	ndhF	lineage
Brassica nigra	DQ200031.1	ndhF	family	*Rorippa palustris*	KC133366.1	ITS	lineage
Braya humilis	AY353124.1	ITS	family	*Rorippa palustris*	JN966508.1	matK	lineage
Braya humilis	KC474211.1	matK	family	*Thlaspi arvense*	HQ593466.1	matK	family
Braya rosea	DQ288743.1	ndhF	family	*Thlaspi arvense*	DQ288839.1	ndhF	family
Cardamine hirsuta	DQ268385.1	ITS	lineage	*Thlaspi caerulescens*	DQ337377.1	ITS	family

*Locus refers to the amplified region, the use of each accession is divided into Family," lineage" and genus, which refer to the three taxonomic levels that were tested.
